# High-Speed Fully Differential Two-Step ADC Design Method for CMOS Image Sensor

**DOI:** 10.3390/s23020595

**Published:** 2023-01-04

**Authors:** Zhongjie Guo, Yangle Wang, Ruiming Xu, Ningmei Yu

**Affiliations:** Department of Electronic Engineering, Xi’an University of Technology, No. 5 Jinhua South Road, Xi’an 710054, China

**Keywords:** CMOS image sensor, differential ramp, time-to-digital conversion, level encoding, two-step

## Abstract

The application requirements of high frame rate CMOS image sensors (CIS) in the industry have not been satisfied due to the speed limitations in traditional single-slope and serial two-step analog-to-digital converters (ADCs). In this paper, a high-speed fully differential two-step ADC design method for CIS was proposed. The proposed method was based on differential ramp and time-to-digital conversion (TDC) technology. A parallel conversion mode was formed that is different from serial conversion, and the robustness of the system was ensured due to the existence of differential ramps. Aiming at the inconsistency between traditional TDC technology and single-slope ADC, a TDC technology based on level coding was proposed. The proposed technology achieves the TDC in the last clock cycle of analog-to-digital conversion, and realized a two-step conversion process at another level. This paper presents a complete circuit design, layout design, and test verification of the proposed design method based on the 55 nm 1P4M CMOS experimental platform. Under the design environment of the analog voltage of 3.3 V, the digital voltage of 1.2 V, the clock frequency of 100 MHz, and a dynamic input range of 1.6 V, this design was a 12-bit ADC with a conversion time of 480 ns, column-level power consumption of 62 μW, differential nonlinearity (DNL) of +0.6/−0.6 LSB, and integral nonlinearity (INL) of +1.2/−1.4 LSB. Furthermore, it achieved a signal-to-noise distortion ratio (SNDR) of 70.08 dB. The proposed design provided a large area array with a high frame rate, and compared with the existing advanced single-slope ADC, its conversion speed increased by more than 52%. It provides an effective solution for the implementation of high frame frequency CIS

## 1. Introduction

The CMOS image sensor (CIS) is gradually replacing the charge-coupled device (CCD) image sensor and playing an increasingly important role in various application scenarios such as autonomous driving technology and artificial vision chips, due to its advantages of high integration, low power consumption, and low cost. At present, the bottleneck that limits the processing speed of CIS lies in the readout conversion stage. The analog-to-digital converter (ADC) is an important part of the CIS readout circuit and the key to the Improvement of CIS performance. In recent years, the research on ADC applied to the CIS field involved a large number of different ADC topologies, such as successive approximation ADC (SAR ADC) [1], σ-δ ADC [2], cyclic ADC (Cyclic ADC) [3], flash ADC (Flash ADC), pipeline ADC (Pipeline ADC) [4], single-slope ADC (SS-ADC) [5], and various combinations of the aforementioned ADC architectures. Considering the trade-off between speed, power consumption, and area, not every ADC structure is suitable for CIS [6,7,8,9,10].

At present, the research on high-speed column-level ADC architecture applied to CIS mainly focuses on SAR ADC, Cyclic ADC, and SS-ADC. The SAR ADC structure was used in [1] with a single conversion time of 2 μs under a 14-bit accuracy. However, due to the use of the capacitive digital-to-analog converter (CDAC) capacitor array, it occupied a large chip area and could not be applied in the CIS with a large area array. The cyclic ADC requires a high slew rate and small area, but there is no effective trade-off between speed and power consumption because it contains a high-speed op amp in each column-level circuit. The cyclic ADC structure was adopted in [3] under a 12-bit precision and 250 MHz main clock frequency. Its conversion time reached 625 ns, and the use of high-speed and high-gain operational amplifiers caused its power consumption to reach 435 μW. On a scale of 100 million, only the power consumption of this ADC structure would be close to 10 W, which limits its application in the CIS of a billion-level area array [7,8,9,10,11,12]. In view of the current research progress and existing problems, based on the traditional SS ADC, this paper proposed a high-speed fully differential two-step ADC structure applied to CIS, which can ensure the power consumption and area advantages of the SS ADC [13,14].

For the incompatibility between TDC technology and ADC technology itself, this paper proposed an analog-time–digital conversion mode based on SS ADC, and the time-to-digital conversion (TDC) technology based on level coding, which completes time-to-digital conversion in the last clock cycle of A/D conversion, and realizes a two-step conversion process at another level. The division of two-step ADC is a general division method for SS ADC. The whole A/D conversion process is divided into a two-step conversion of coarse conversion and a two-step conversion of fine conversion. The dynamic range of the coarse conversion includes the dynamic range of the entire ADC, and the dynamic range of the fine conversion is a step value of the coarse conversion. In this paper, double ramp and TDC were nested on the basis of two-step transformation.

Through verification and simulation, the ADC conversion speed was increased by more than 52%, compared with the existing advanced single-slope ADC. The proposed method had the following three innovation points:

(1) Symmetrical design: The complete differential circuit structure not only completes the high-speed conversion process, but also ensures the stability of ADC circuit;
(2) Under this premise, the differential ramp is nested in a two-step SS ADC. Compared with the traditional SS ADC, the parallel data conversion idea similar to the time interweaving, the time utilization rate of this design is increased by more than 50%, which can complete the faster analog-to-digital conversion process;
(3) TDC technology based on level coding is proposed, which completes T/D conversion in the last clock cycle of A/D conversion to achieve another level of two-step conversion process. The introduction of TDC technology greatly improves ADC’s conversion rate, and the TDC encoding method is improved, so that the matching degree with ADC is higher. With the introduction of N-bit TDC technology, the conversion speed of SS ADC is increased by 2^N^ times at the same precision level. Due to the limitation of clock frequency, n cannot be increased indefinitely. Therefore, this paper uses 2-bit TDC on the premise of ensuring the feasibility of the actual conversion process.

This paper focuses on analyzing the realization principle of the two-step ADC, and presents circuit design and comprehensive parameter testing on the experimental platform.

## 2. CIS System Architecture

**Figure 1:**
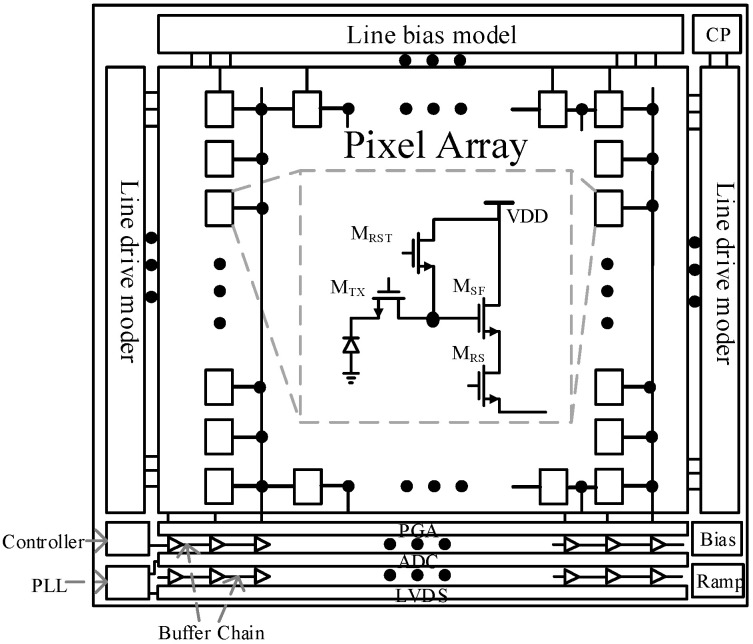
Schematic diagram of the CIS system framework.

The overall architecture of a CIS specifically includes a pixel array, a row driving module, a column biasing module, a readout circuit, a control circuit, a clock generator, a ramp generator, and a few driving circuits [15,16]. Figure 1 shows the overall architecture of the CIS, in which the pixel array completes the photoelectric signal conversion. The obtained electrical signal is amplified, sampled, and converted by the readout circuit. The clock signals generation circuit and controller, as well as the column bias module and the row drive module, provide timing control and analog bias for the pixel unit and the readout circuit to complete the image processing and readout together. The current bottleneck restricting the processing speed of CIS lies in the readout conversion stage irrespective of the current mainstream rolling shutter exposure or global exposure mode. The ADC is a key part that affects signal conversion processing. Therefore, a high ADC accuracy must be guaranteed in order to ensure imaging quality [17,18,19]. However, the time required to complete the N-bit conversion for the conversion mode of SS ADC is as follows:

(1) $${\;T}_{\mathrm{conversion}}=T_{\mathrm{CLK}}\times 2^{N}$$

Equation (1) shows that every time the conversion accuracy of the SS ADC improves by 1-bit, its speed will decline exponentially. Therefore, the core of improving the SS A/D conversion technology is mainly focused on structure-related optimization and innovation, and it will provide a breakthrough in the subsequent development of CIS.

## 3. Principle Analysis of Fully Differential Two-Step Design

The division of two-step ADC is a general division method for SS ADC. The whole A/D conversion process is divided into a two-step conversion of coarse conversion and a two-step conversion of fine conversion. The dynamic range of the coarse conversion includes the dynamic range of the entire ADC, and the dynamic range of the fine conversion is a step value of the coarse conversion. In this paper, double ramp and TDC are nested on the basis of two-step transformation.

The conversion principle of SS ADC is divided into two steps: analog-to-time conversion (ATC), and time-to-digital conversion (TDC). In the circuit architecture of SS ADC, the minimum voltage value that the structure can recognize over the entire dynamic range is a variable of the ramp signal within a clock cycle. If the ramp is compared to a ruler, the variable generated on the ramp is the indexing value of the ruler. Therefore, the ramp signal can be equivalent to the equivalent rising ramp Vramp1 or the equivalent falling ramp Vramp2 generated by summing the increment or decrement of the ramp signal. From the perspective of ATC, the comparator directly maps the time relationship of the input signal on the ramp signal in order to complete the transition from the analog signal domain to the time signal domain. The subsequent digital counting logic maps this time relationship to complete the transition from the time domain signal to the digital domain signal. The final output digital code completes the two-step conversion process of high M-bit coarse conversion and low N-bit fine conversion.

**Figure 2:**
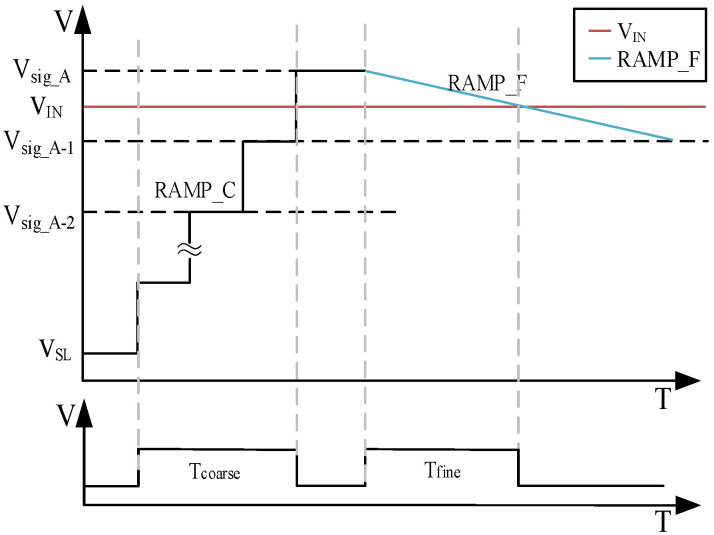
Schematic diagram of normal conversion.

In Figure 2, RAMP_C represents coarse ramp during coarse conversion; RAMP_F represents fine ramp during fine conversion; V_IN_ represents input signals to be quantified; T_coarse_ conversion time; T_fine_ represents fine conversion time; V_sig_A-n_ represents the size of the step value of each coarse ramp; and V_SL_ represents the initial value of the coarse ramp.

Each input ramp step value of the SS ADC corresponds to the digital counting logic. For M-bit coarse conversion, RAMP_C is divided into 2^M^ coarse steps, and the conversion time of each coarse step corresponds to one clock cycle T_CLK_. The time required to complete the coarse conversion is:

(2) $${\;T}_{\mathrm{coarse}1}=2^{M}\times T_{\mathrm{CLK}}$$

The conversion time of each coarse step is still T_CLK_ at this time due to the introduction of the differential coarse ramp. Unlike traditional serial two-step SS ADC, the dynamic range of coarse conversion is divided into two parts, and half of the coarse conversion time becomes redundant time. The time required to finally complete the coarse conversion is as follows:

(3) $${\;T}_{\mathrm{coarse}2}=2^{M-1}\times T_{\mathrm{CLK}}$$

For the fine phase, the same comparator that was triggered in the coarse phase must be used. So, the fine phase uses single comparator only. This means that the full ramp swing would be used. As far as the N-bit refinement is concerned, RAMP_F is divided into 2^N^ fine steps. The conversion time of each fine step corresponds to one clock cycle T_CLK_ and, therefore, the time required to complete the fine conversion is:

(4) $${\;T}_{\mathrm{fine}}=2^{N}\times T_{\mathrm{CLK}}$$

Considering the general situation and assuming M-bit coarse conversion and N-bit fine conversion, the time required for a two-step conversion of a nested differential ramp is as follows:

(5) $${\;T}_{\mathrm{final}}=\left(2^{M-1}+2^{N}\right)\times T_{\mathrm{CLK}}$$

The advantages of the fully differential two-step conversion mode proposed in this paper are as follows: (1) A parallel conversion mode is formed that is different from serial conversion, and at the same time, the consistency and robustness of the system are ensured thanks to the existence of the differential ramp. (2) Compared with the traditional two-step SS ADC, the proposed structure results in a speed gain of more than 50%, and this speed gain is more obvious in the higher-precision conversion process.

## 4. TDC Technology Based on Level Coding

The two analog voltages that were converted, A and B, describe an extreme case in which the two input signals form an analog interval where the same digital output is obtained. If the signal was greater than the analog voltage B that was converted, the digital output of the ADC increased by one unit, and if the signal was less than the analog voltage A that was converted, the digital output of the ADC decreased by one unit. When there was an input signal in the AB interval, one method to obtain a more accurate conversion result was to use a higher-precision TDC globally with a high-frequency clock and a higher-bit counter to reduce the minimum resolution time of the ADC. Another method used more precise local conversion, i.e., the difference between the signal and Time2 was quantized with a higher precision TDC within the clock cycle of the signal. Since the ATC conversion used the comparator to complete the time mapping of the input signal on the ramp signal, the above method uses a higher-precision TDC conversion to measure the difference between the comparator inversion time and the end time of the current clock cycle [20].

**Figure 3:**
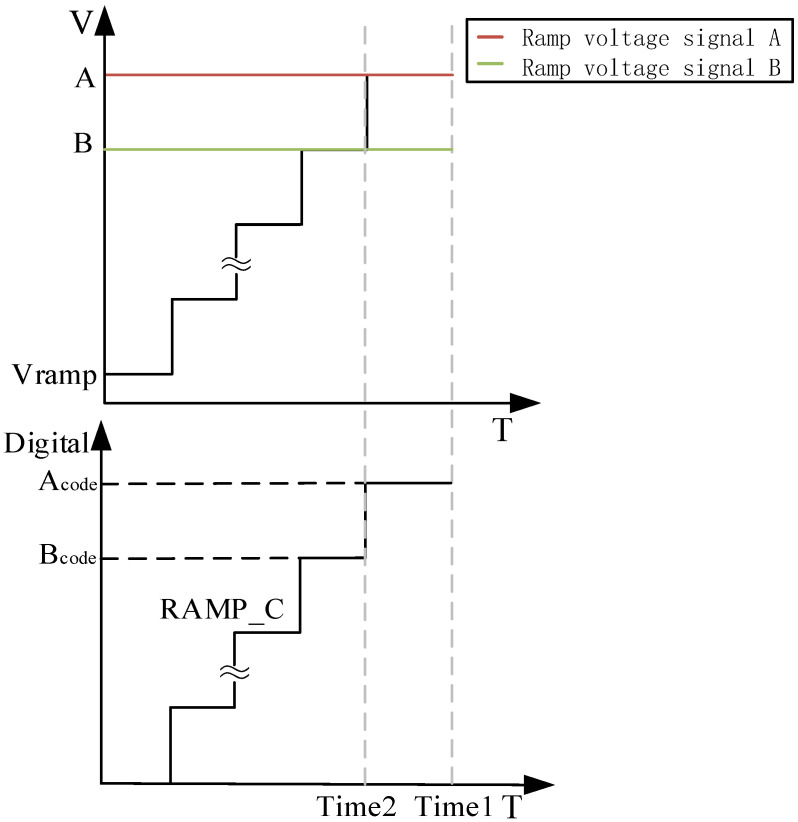
Schematic diagram of time difference conversion.

In Figure 3, Vramp represents the ramp signal; A and B represent the voltage values of a specific ramp; RAMP_C represents the coarse ramp; and A_code_ and B_code_ represent the numerical codes corresponding to the voltage values of the two ramp signals A and B, respectively.

**Figure 4:**
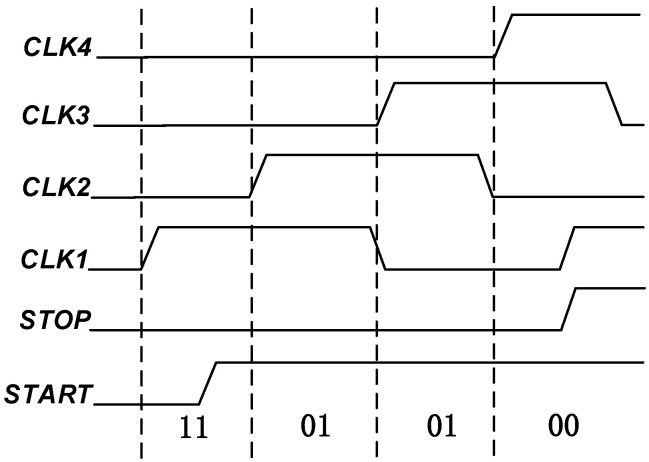
Schematic diagram of traditional clock compression TDC coding principle.

Figure 4 shows the coding principle of a traditional TDC. Under this coding logic, the sequential arrival of the four clocks requires four flip-flops to be triggered in sequence. The outputs of the flip-flops flip when different clock edges arrive. Through this edge-triggered method, different output results are then passed through the state transition diagram. The encoder is used to encode different output results, and the 2-bit binary digital conversion result of 00~11 is obtained.

**Figure 5:**
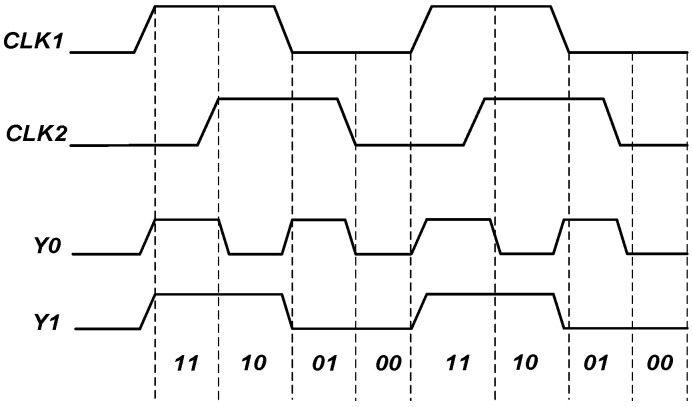
Schematic diagram of the principle of TDC level coding.

Figure 5 shows the level coding principle proposed in this paper. Two clocks, CLK1 and CLK2, were provided by the delay-locked loop (DLL), which corresponded to different combinations of high and low levels at different conversion moments in a clock cycle. CLK1 and CLK2 were passed through the digital logic gate and D flip-flop to output the 2-bit binary digital conversion results Y0 and Y1 of 00~11 in one clock cycle.

In addition, because edge coding requires D triggers for edge judgment, in 2-bit TDC, not only were four clock edges needed, but also at least four D triggers were needed for edge coding. However, the level coding method in this paper only requires less than twice D triggers to realize interval division. This area advantage is very obvious in the application of CIS of super-large array.

**Figure 6:**
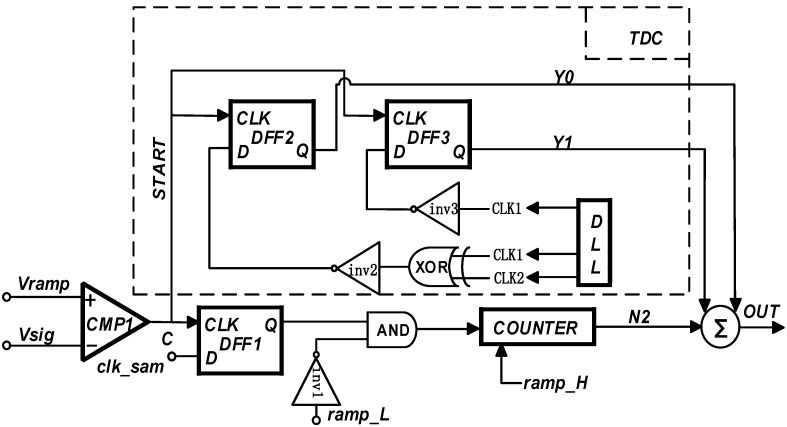
Schematic diagram of TDC coding circuit architecture.

Figure 6 shows the two-step high-speed SS ADC circuit based on the level-encoded TDC. The advantages of this circuit are as follows:

① It only needs two clocks provided by the DLL, and uses the levels of the two clocks for digital encoding. In this encoding method, the TDC’s demand for the DLL is more effectively released, and at the same time, the problem of low conversion accuracy caused by the clock jitter of the DLL is improved;
② The traditional edge coding requires complex coding logic, while the proposed TDC structure coding method is relatively simple. The delay between input and output is smaller, and the conversion speed is faster. The proposed structure can complete the analog-time–digital high-speed conversion process while ensuring the conversion accuracy, and provides an effective solution for the high-performance CIS;
③ In this regard, due to the low circuit architecture sacrifice coding design, in addition to the advantage of dynamic power consumption, the signal delay in the system is lower. In the 55 nm process, the delay time of two D-triggers is saved. Since the delay time is at the ps level, the speed benefit is only a small part compared with the above advantages.

## 5. Fully Differencial SS ADC Design

**Figure 7:**
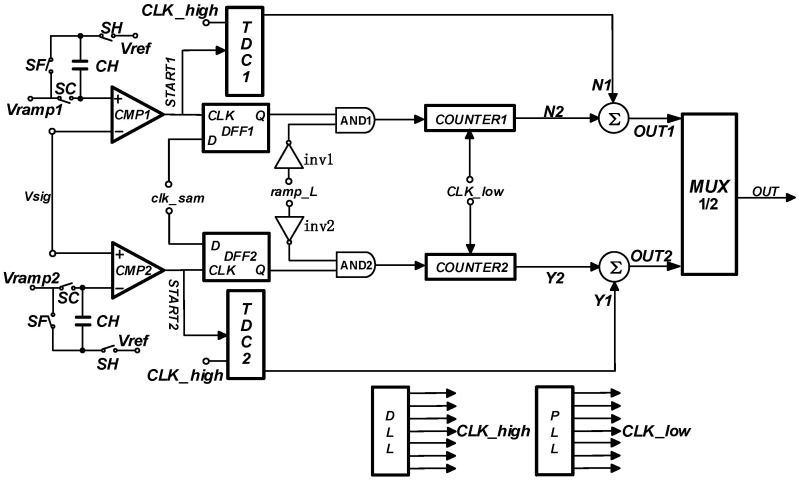
Schematic of the proposed circuit.

The fully differential circuit architecture is shown in Figure 7. The circuit is composed of a sampling circuit, comparator, TDC, and digital counting logic in the full differential form. The final output of the conversion result is achieved by the data selector. The low-frequency clock used by the digital counting logic is provided by the phase-locked loop PLL, and the equivalent high-frequency clock used by the TDC is provided by the delay-locked loop DLL. The ramp generator generates ramp signals Vramp1 and Vramp2 in differential forms, and the photoelectric conversion signal Vsig is input from the sampling circuit to complete the system. The high-speed fully differential two-step ADC proposed in this paper divides the 12-bit conversion process into 5-bit coarse conversion, 5-bit fine conversion, and 2-bit TDC conversion processes. 

It is worth noting that in the two stages of coarse conversion and fine conversion, CMP1 and CMP2 are used to compare the two ramps with Vsig at the same time. Then, since there must be an intersection between one ramp and Vsig, the comparator is used to judge and complete the turnover process. The corresponding counter counts and finishes the signal that has completed the correct judgment. After the coarse conversion is completed, the upper plate of capacitor CH stores the signal value of a certain coarse step corresponding to Vsig. On this basis, the double ramp of the fine conversion is input into two comparators at the same time, and the above signal process is repeated. Since the precision of the coarse conversion and the precision of the fine conversion designed in this paper is both 5-bit, the above signal process is repeated. Therefore, the counter of the branch can continue to complete the counting process of the fine conversion after the completion of the coarse conversion counting and storage. For the two processes of coarse and fine conversion, the count of the counter can be carried out synchronously with the arrival of each CLK. That is to say, the counting results of coarse and fine conversion can be added and subtracted within the counter under the same bit.

After the integration of the coarse and fine counting results, the count results can be subtracted from the count results of TDC to obtain more accurate counting results. Finally, the binary data selector can judge the correct results and output them. Because one branch is not flipped and will be counted in full, MUX only needs to complete the selection of the correct count results and the wrong full count results for the two branches.

First, coarse conversion is performed. In the switched capacitor control circuit, the control switches SC and SH are turned on, and Vramp1 and Vramp2 are the differential coarse ramp voltages at this time, starting from 0 and stepping to the full swing of the coarse ramp voltage VFS. Each step value is a step ΔC of the coarse ramp voltage. The comparator compares the ramp input signal with the signal to be converted Vsig. The coarse ramp voltage increases by ΔC in every step. If after m steps, the output of the comparator becomes a high level, this indicates that the input signal is:

(6) $$\;m\Delta C<\mathrm{Vsig}<\left(m+1\right)\Delta C\;$$

In this coarse conversion, the interval where Vsig was located is found. At this time, the switch SH was turned off, and the capacitor CH stored the coarse ramp voltage value (m + 1)ΔC at this time. The voltage difference between the upper and lower plates of the capacitor CH was (m + 1)ΔC-Vref, where Vref was a fixed level. After the coarse ramp voltage steps to the full swing voltage VFS, the switch SC was turned off and the coarse conversion process ended.

Second, the refinement operation was carried out. At this time, the switch SF was in the on the state in the switched capacitor circuit, and Vramp1 and Vramp2 were differentially thin ramp voltages, defined as VR, which were connected to the lower pole of the capacitor CH in the switched capacitor circuit. As the capacitor CH stored the previous coarse ramp voltage value (m + 1)ΔC, the voltage value VC of the ramp input terminal of the comparator was VR + (m + 1)ΔC. The fine ramp voltage VR changed stepwise from −ΔC to Vref, where the step value of each time was ΔF of the fine ramp voltage. The VC changed from mΔC to (m + 1)ΔC, i.e., the fine conversion was performed on the coarse conversion interval where VIN was located. The comparator compared VC with the signal to be converted Vsig. If the thin ramp voltage VR goes through n steps, the output of the comparator reaches a high level, indicating that the input signal is as follows:

(7) $$\;m\Delta C+\left(n-1\right)\Delta F<\mathrm{VIN}<m\Delta C+n\Delta F\;$$

Third, a 2-bit TDC process was performed. The converted end signal was used as the start signal of the TDC. At this time, the clock edge of the low-frequency clock of the fine ramp counter was used as the end signal of the TDC. The configuration can be completed in the SS ADC through the periodic encoding process. The equivalent high-frequency clock required by TDC encoding was provided by the DLL. At the same time, the counter adopted a simple chain structure in order to have a strong adjustment space, and the bottom flip-flop adopts a D flip-flop that can be reset and set synchronously. The reset weight was designed to be higher than the set weight, and the initial value could be configured with the NAND gate circuit. By setting the initial value to −1, the process of subtracting the TDC count result from the fine-converted count result was omitted. The actual count became the coarse and fine-converted count resulting in the form of a high 10-bit digital code. The inverted code was directly used as the low 2-bit digital code, and the 12-bit analog-to-digital conversion result is directly completed. Consequently, the column-level ADC circuit reduced the area and power consumption of the subtractor circuit. Last, a complete conversion cycle ended within this fine conversion interval.

Since the precision of coarse conversion designed in this paper was 5-bit and the precision of fine conversion was also 5-bit, in terms of time, for the two processes of coarse and fine conversion, the count of the counter can be carried out synchronously with the arrival of each CLK. That is to say, the counting results of coarse and fine conversion could be added and subtracted within the counter under the same bit. Then it was output by the register inside the counter.

**Figure 8:**
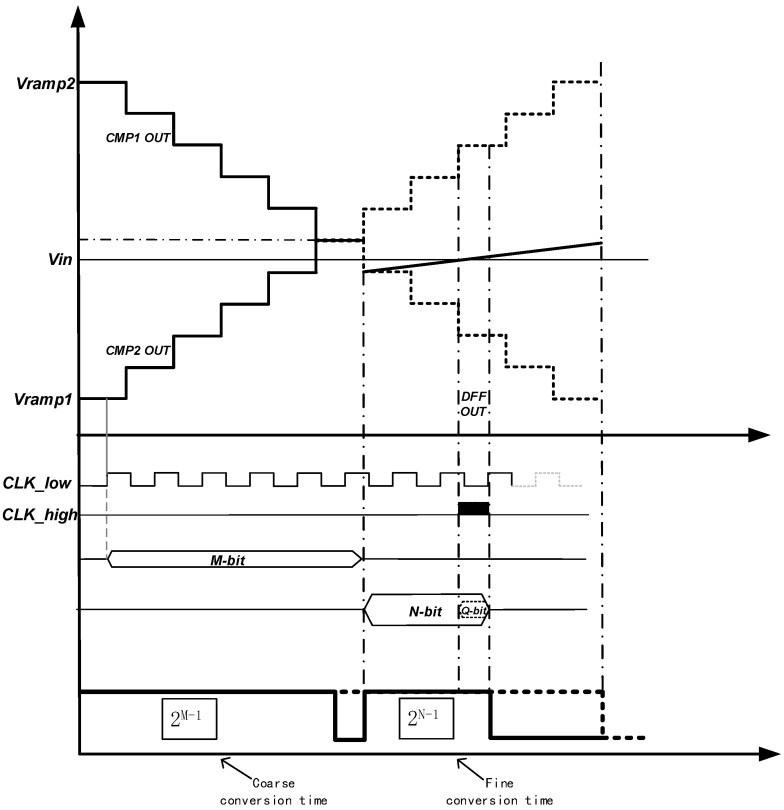
Timing diagram of the proposed circuit.

As Vin is a signal to be converted, it does not change. As shown in Figure 8, the completion moment of the fine conversion stage is ideally the intersection of Vin and the fine ramps, and the final end moment is the end moment of the fine ramps. However, since the SS ADC architecture itself is a digital code sampling by a counter, the minimum resolution of the counter is controlled by the master clock CLK, so the actual end time is the next edge of the clock after the Vsig intersects the ramp. In addition, it is worth noting that in the last period of time before the clock edge arrived, TDC technology was inserted in this paper to complete the conversion process at a higher speed with the same accuracy level.

For the description in Figure 8, the final conversion time is only the end of the fine conversion time marked by the solid line. The ramp full swing is only to highlight the speed advantage of this design compared with other SS ADC in the conversion process, which is one of the innovations of this paper. In practical application, the ramp generator only needs to generate ramps with half the dynamic range.

Figure 8 shows the working sequence of the ADC circuit based on differential ramp and TDC. The differential ramp is composed of M-bit coarse ramp and N-bit fine ramp, the TDC is Q-bit, and the guaranteed total conversion accuracy is under the premise of (M + N + Q) bits. The final actual conversion time is only $2^{M-1}+2^{N}$. Compared with the aforementioned traditional two-step ADC, it can achieve a higher-speed conversion, which provides an effective solution for the realization of CIS with a high frame rate and a large area array.

## 6. Experimental Results and Data Analysis

**Figure 9:**
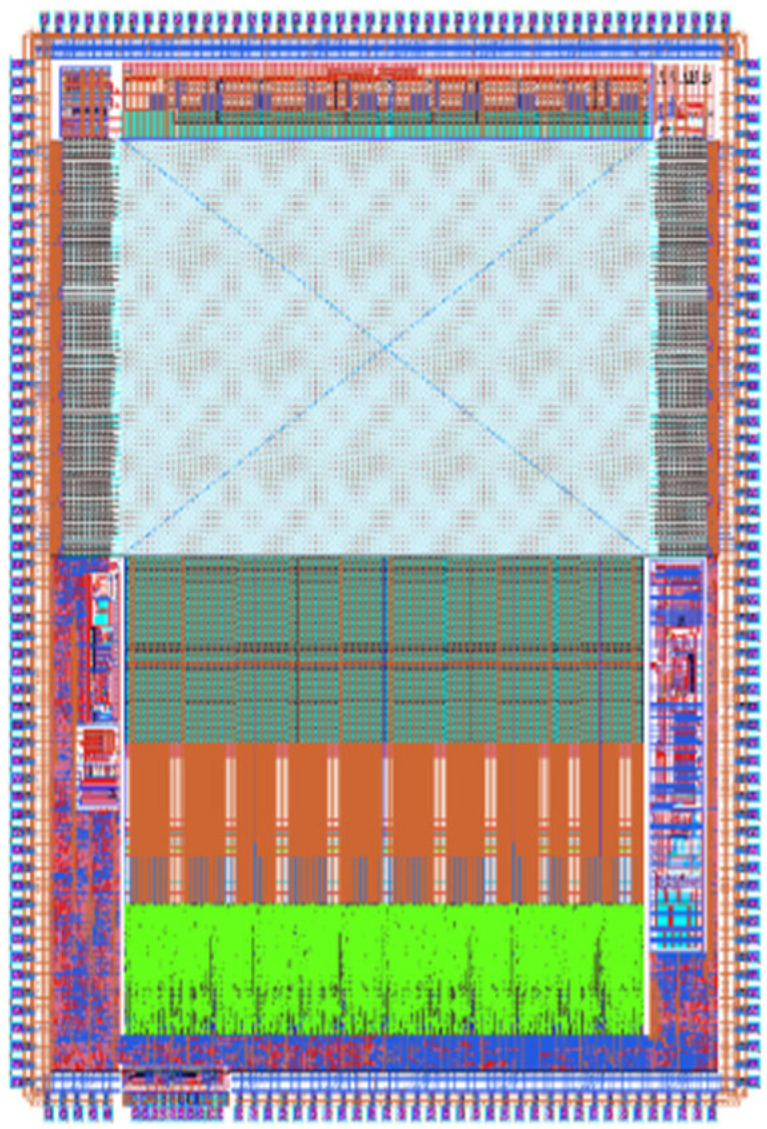
CIS chip overall simulation platform.

According to the design principle and specific circuit design of high-speed fully differential two-step ADC based on differential ramp and TDC technology, an experiment was carried out to verify the feasibility of the proposed method. The verification platform for the CIS with a scale of 8192 × 8192 based on a 55 nm 1P4M process was used for testing of results and data analysis as shown in Figure 9. The ADC had a 12-bit conversion accuracy, the analog and digital power supplies were equal to 3.3 and 1.2 V, respectively, and the clock signal frequency was 100 MHz.

In the performance verification phase, the ADC was verified on a 50 × 50 mm large array CMOS image sensor platform, which consisted of 8192 × 8192 pixel array. The ADC conversion accuracy was 12 bit, the analog, and digital power supplies were 3.3 and 1.2 V, respectively, and the clock signal frequency was 100 MHz. The deviation between the simulation results and the actual stream chip results was less than 10% after multiple tests on the experimental platform. Therefore, the author described in this paper the simulation results considering the influence of “parasitism”, “random noise”, and other factors, rather than just the sorting of simulation data. At the same time, the main feature of this ADC was its high-speed application in CIS. The simulation results in terms of conversion rate were basically consistent with the theoretical analysis. To this extent, the comparison between literatures should still be convincing. It is worth noting that the ADC design technology only stays in the simulation stage of the experimental platform due to the matching problem between it and other modules. To some extent, this design method provides a feasible implementation scheme for the future large array high frame rate CMOS image sensor, which is the practical research significance of this paper. Figure 9 shows post-simulation platform. The ADC part of the simulation platform is composed of ADC layout multiplexing units in the paper.

**Figure 10:**
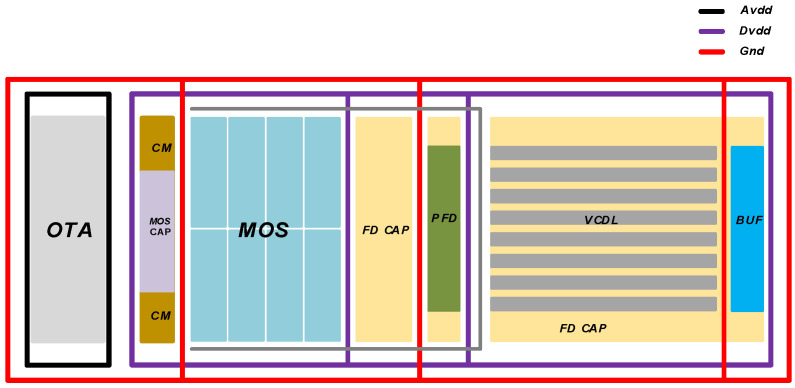
ADC overall layout design.

**Figure 11:**
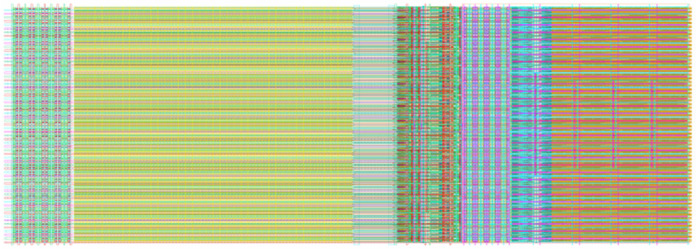
ADC overall layout.

Figure 10 and Figure 11 show the column multiplexing unit obtained by calling each module and splicing each functional circuit composed of analog and digital units. The analog and digital modules use a group format to supply power to each unit, respectively, and a large number of FD capacitors are placed in the vertical gap. To ensure the resistance of the power supply to digital noise, the digital and analog circuits adopted a separate design method and independent substrate to avoid the crosstalk caused by digital noise.

Due to the design characteristics of multiplexing, the column unit signal transmission path and interface information were consistent with the multiplexing unit. The analog bias signal provided a bias to all the column circuits in the array through the reserved wiring channel. All the analog signals were shared by the column circuits, except the current bias which is set independently by each multiplexing unit. The current bias channel and the analog signal channel were designed separately, and 8192 lines were used to independently provide bias to each multiplexing unit. All digital signals formed an array-level drive chain area through the drive units designed in the multiplexing unit, and provide control signals to all column circuits. The signal delay in the driver chain was about 10 ps, which was considerably less than the system clock period. As the 2-phase clock required for TDC has a similar transmission path to the clock signal in the array transmission, it can still be ensured that the multi-phase clock was used to divide the interval of the fixed clock period in the TDC process. In addition, each functional circuit of the ADC was constrained by the clock signal. Therefore, at a signal delay of the order of 8192, the control signal of any column of the ADC array was consistent with the clock signal. The ADC array based on this design principle had a high tolerance for the control signal delay. Due to the multiplexing design characteristics, the performance of the 1024 array was consistent with that of the column multiplexing unit.

Figure 10 is the layout design scheme completed considering the practical application of the circuit in this paper. Under the experimental platform of 55 nm process, OTA was the operational amplifier used for signal sampling of the experimental platform, FDCAP was the filter capacitor, CM was the comparator, MOS CAP was the switching capacitor circuit of the sampling part, and MOS was the digital logic part. Subsequently, the DLL part of the delay clock was provided for TDC. PFD was the charge pump, VCDL was the voltage controlled delay chain, and BUF was the signal driver chain.

Among CMOS image sensors, SS ADC mainly brings column fixed pattern noise (CFPN) and CFPN spatial noise. On the one hand, it can be eliminated by digital correlation double sampling. On the other hand, by optimizing the layout to ensure the matching, the symmetry design in this paper can be better in terms of matching. Therefore, this structure can be solved to a certain extent for CFPN.

**Figure 12:**
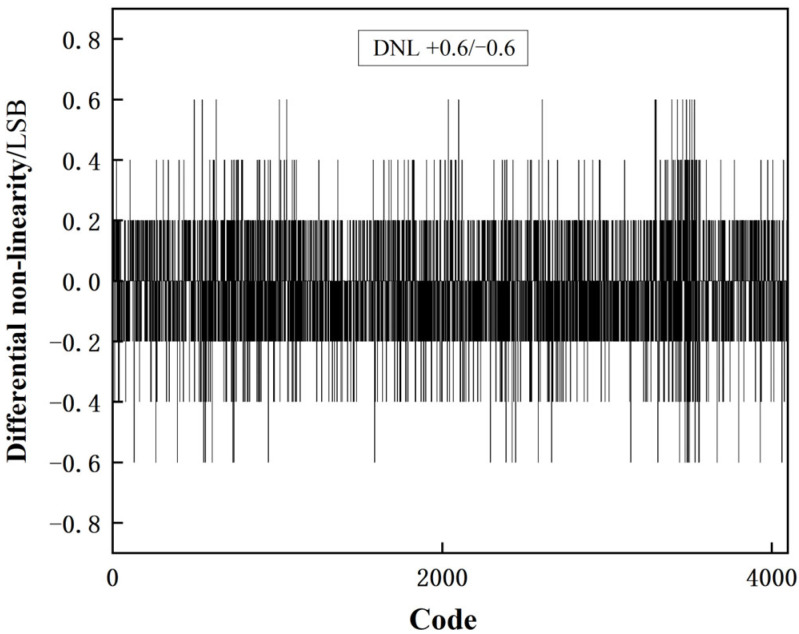
DNL test result.

**Figure 13:**
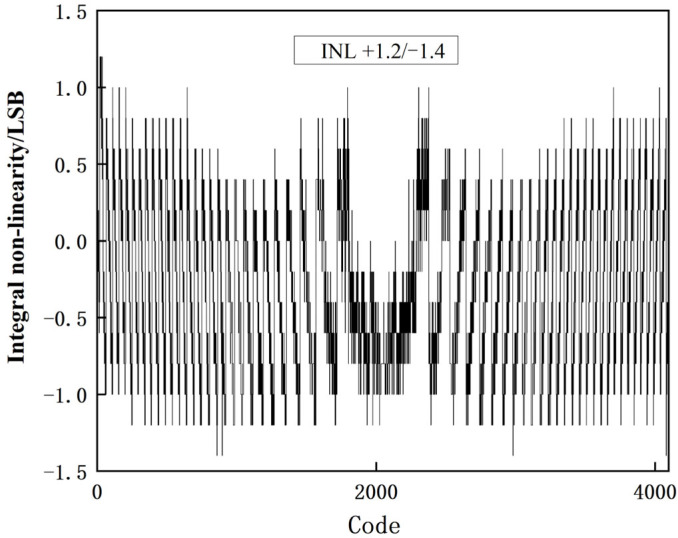
INL test result.

Figure 12 and Figure 13 show the static verification results of this experiment. The experiment is performed with an observation accuracy of 0.2 LSB. The obtained differential nonlinearity (DNL) and the integral nonlinearity (INL) are shown in Figure 12 and Figure 13, respectively, where the DNL was + 0.6/−0.6 LSB and the INL was +12/−1.4 LSB.

**Figure 14:**
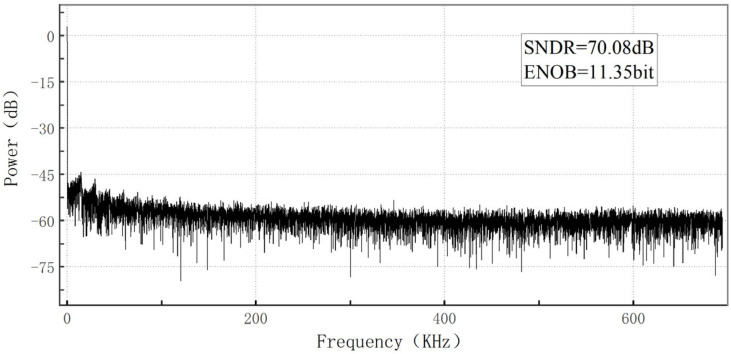
Signal to noise ratio analysis.

Figure 14 shows the results of the FFT analysis of 8192 groups of data sampled at 147.964 Hz signal with a sampling frequency of 1.21 MHz. Transient noise was added to the simulation, and the noise frequency was 1~1 GHz. Inputting the sine wave, the sine wave frequency was 1/2 of the sampling frequency, sampling quantization of sine wave, and then use digital coding to reconstruct sine wave, the reconstructed sine wave FFT analysis.

The values of signal-to-noise-distortion ratio (SNDR), the effective number of bits (ENOB), column-level power consumption, and dynamic range of the ADC designed in this paper are equal to 70.08 dB, 11.35 bits, 62 μW, and 1.6 V, respectively.

Table 1 compares the results obtained by the design method proposed in this paper and the existing cutting-edge research. The ADC architectures in the table are all compared under the accuracy of 12-bit. References [6,7,8] are all two-step structures with coarse and fine conversion. Compared with [6], the power consumption of the proposed design is reduced by 14% and the conversion speed is improved by 95.2%. Compared with [7], the power consumption of the proposed design is reduced by 45% and the conversion speed is improved by 92.5%. Compared with [8], the power consumption of the proposed design is 32% higher but it provides about 62.5% speed gain, and the static characteristics are better under the same process conditions.

Reference [9] is a two-step architecture combining SS ADC and TDC. Compared with [9], the power consumption of the proposed design was reduced by 65%, and the conversion speed was improved by 52%. The above comparison shows that under the same accuracy, the ADC designed in this paper has obvious speed advantages compared with the current cutting-edge architecture, and the conversion speed increases by more than 52% on the premise of ensuring low power consumption. Due to the advanced technology and system architecture of the ADC designed in this paper, the actual speed improvement is basically consistent with the theoretical analysis. It can be observed that the design method proposed in this paper has obvious advantages compared with the existing references [6,7,8,9].

## 7. Conclusions

Aiming at the common speed bottleneck problem of traditional SS ADC and serial two-step ADC, this paper proposed a high-speed fully differential two-step ADC design method for CIS. The method was based on the differential ramp and TDC technology, which nested differential conversion in a two-step conversion, forming a parallel conversion mode that was different from serial conversion. Meanwhile, the time-to-digital converter was inserted into the SS ADC. Under the conditions of completing the quantification with the same accuracy, the TDC conversion process was completed at the same time in the final stage of the ADC conversion readout, which greatly reduced the time overhead and enabled the ADC to achieve a higher time utilization rate, and finally completed the ultra-high-speed analog-to-digital conversion process. Compared with the existing two-step SS ADC, the conversion speed of the proposed design method increased by more than 52%. This time gain became more obvious with the improvement of accuracy.

## Figures and Tables

**Table 1:** Performances compared with state-of-the-art methods.

Reference	[6] (Simulation)	[7]	[8] (Simulation)	[9]	This Work
**Structure**	TS-SS	TS-SS	PTS-SS	SS-TDC	TS-SS
**Precision (bit)**	12	12	12	12	12
**Conversion range (V)**	1.2	-	1.472	-	1.6
**Conversion time**	10 μs	6.38 μs	1.28 μs	1 μs	480 ns
**DNL (LSB)**	0.76/−0.8	+1.34/−0.49	+0.8/−0.8	+1.1/−0.4	+0.6/−0.6
**INL (LSB)**	1.06/−0.84	+2.44/−2.47	+2.1/−3.5	+5.8/−8.2	+1.2/−1.4
**ENOB (bit)**	11.25	-	11.33	-	11.35
**Power (μw)**	72	112.5	47	177	62

## Data Availability

The data presented in this study are available on request from the corresponding author. The data are not publicly available due to privacy.
